# Improving Poly(3-Hydroxybutyrate) Properties Using Nanocellulose in Biomedical Applications: Thermal, Mechanical and Biological Studies

**DOI:** 10.3390/ijms26199795

**Published:** 2025-10-08

**Authors:** Karolina Maternia-Dudzik, Łukasz Ożóg, Zuzanna Bober, Rafał Oliwa, Mariusz Oleksy, Angelika Kamizela, Agnieszka Szyszkowska, Katarzyna Rafińska, Weronika Gonciarz, Kamil Gancarczyk, Anna Czerniecka-Kubicka

**Affiliations:** 1 Department of Microbiology, Faculty of Medicine, University of Rzeszow, 35-959 Rzeszow, Poland; kmaternia@ur.edu.pl; 2 Department of Pharmaceutical Technology and Medical Physics, Faculty of Medicine, University of Rzeszow, 35-959 Rzeszow, Poland; lozog@ur.edu.pl (Ł.O.); zbober@ur.edu.pl (Z.B.); angelikakamizela@gmail.com (A.K.); 3 Department of Polymer Composites, Faculty of Chemistry, Rzeszow University of Technology, 35-959 Rzeszów, Poland; oliwa@prz.edu.pl (R.O.); molek@prz.edu.pl (M.O.); 4 Provincial Hospital in Kielce, 25-736 Kielce, Poland; szyszkowska.agnieszka@gmail.com; 5 Department of Environmental Chemistry and Bioanalytics, Faculty of Chemistry, Nicolaus Copernicus University in Torun, 87-100 Torun, Poland; katraf@umk.pl; 6 Department of Immunology and Infectious Biology, Faculty of Biology and Environmental Protection, Institute of Microbiology, Biotechnology and Immunology, University of Lodz, 90-237 Lodz, Poland; weronika.gonciarz@biol.uni.lodz.pl; 7 Department of Material Science, Faculty of Mechanical Engineering and Aeronautics, Rzeszow University of Technology, 35-959 Rzeszow, Poland; kamilgancarczyk@prz.edu.pl

**Keywords:** nanocomposites, poly(3-hydroxybutyrate), nanocrystalline cellulose, biocompatibility

## Abstract

Poly(3-hydroxybutyrate), P3HB, is a biodegradable polymer produced and stored by different bacterial strains, including *Ralstonia eutropha H16.* P3HB was used to prepare biocompatible composites modified by nanocellulose. This study aimed to assess selected thermal, mechanical, and biological properties of the obtained nanobiocomposites. Thermal properties, as determined by differential scanning calorimetry measurements, were established. The crystallinity of nanocomposites and polymeric matrix was investigated using DSC analyses. The morphology of the nanocomposites was evaluated using scanning electron microscopy. The Food and Drug Administration and the European Medicines Agency confirmed the immunosafety of the tested nanocomposites and noted they had either no or very low levels of endotoxin contamination. Some mechanical properties of the investigated materials were also measured and are presented here. It was estimated that the addition of 1% by mass of nanocrystalline cellulose to P3HB causes the greatest improvement in the plasticization of the material, characterised by the best processing and utility properties. The processing window of nanobiocomposites was extended by approximately 25 °C in reference to the unfilled poly(3-hydroxybutyrate). Mechanical and thermal tests revealed that the most desirable properties oscillate around the addition of 0.5% and 1% nanocrystalline cellulose by mass in the nanobiocomposites. Biological studies on implant applications have shown that the addition of only 0.5% nanofiller to a nanobiocomposite can be of key importance.

## 1. Introduction

Polyhydroxyalkanoates (PHAs) are biodegradable polymers made by many microorganisms as stored forms of carbon and energy inside cells. Recent efforts have been made to improve PHA production through genetic manipulation and optimisation of fermentative conditions, as demonstrated by García (2024) in the utilisation of inexpensive starting materials and novel cultivation methods to enhance PHA yield, particularly in biomedical applications [1]. Mixing of PHAs with other biodegradable polymers has become a popular approach to decrease production costs whilst improving material properties. This strategy aims to render PHAs more competitive with oil-based plastics and to extend their potential usage [2], as seen in the work by [3] on the optimisation of operating conditions in phototrophic mixed culture (2019) [3]. The true revolution here is a method that utilises light and organic waste to synthesise biomaterials [3] in a sustainable manner. Ongoing work on synthesis methods and improving the properties of PHA-based materials underscores the growing interest in these materials as a sustainable alternative to conventional plastics. Ongoing research aims to enhance their production efficiency and expand their applications.

Poly(3-hydroxybutyrate) (P3HB) is the most extended PHA. It is a polyester derived from 3-(*R*)-hydroxybutyric acid, characterised by biodegradability in both aerobic and anaerobic environments, as well as biocompatibility; the human body tolerates it well. Its degradation products, such as 3-hydroxybutyrate, are naturally present in human metabolism, which minimises the risk of undesirable immunological reactions. Its compatibility with biological systems and its natural biodegradability make it a preferred choice for medical uses, including drug delivery, tissue engineering scaffolds, and dissolvable surgical sutures [4,5,6,7,8]. The polymer can be produced from common, easily accessible substrates and renewable resources. Its thermoplastic characteristics are comparable to those of petrochemical-based plastics like polyethene polypropylene. In addition, P3HB is produced naturally as a reserve substance in the fermentation process of sugars and fats by microorganisms such as *Ralstonia eutropha*, *Zoogloea ramigera*, *Escherichia coli*, *Alcaligenes* species, *Methylobacterium* species, *Pseudomonas* species, and *Azetobacter* species, and some of them, e.g., *Azohydromonas lata*, recombinant *Escherichia coli*, and *Cupriavidus necator*, are successfully used for the production of P3HB on an industrial scale [6,9,10,11,12,13,14]. The method of storing carbon and energy is crucial for the affordable use of P3HB for medical purposes, given its production from renewable resources and its synthesis by various microorganisms. This practice not only reduces production costs but also meets the growing demand for environmentally friendly materials in the healthcare sector. In addition, P3HB exhibits the property of shape memory and is therefore classified as an intelligent material. Shape memory materials can regain their original form under the influence of a stimulus such as energy in the form of heat, light, magnetic and electric fields, ultrasound, or chemicals [15]. Furthermore, P3HB’s flexibility makes it ideal for manufacturing various medical devices such as sutures, meshes, implants, and tissue engineering scaffolds, while also boosting its cost-effectiveness [16,17]. Biocompatibility, biodegradability, and cost-effectiveness make P3HB an essential material for medical applications, providing sustainable alternatives to conventional petroleum-based polymers. Zeneca Bioproducts has developed a fermentation process that produces PHA copolymers. In this group, P3HB and poly(3-hydroxybutyrate-co-3-hydroxyvalerate), PHBV, known under the trade name Biopol^®^(Zeneca Bioproducts, Billingham, UK), are produced on an industrial scale. Such biopolyesters are used in medicine to produce coatings for controlled-release tablets, for the production of bone implants, and in tissue engineering. Their share in the medical and healthcare market is estimated at 97,400 tons [18].

To further enhance its performance, P3HB is often modified with various additives, resulting in polymer composites. The primary aim of treating P3HB with fillers is to improve its physical, mechanical, and thermal properties, as well as the functionality targeted for specific applications [19,20,21]. Such an approach serves as a cure for a weak link in P3HB, which is too fragile in some medical technologies. P3HB synthesised in cooperation with auxiliaries, e.g., plasticizers, nanomaterials, and natural fibres significantly extends not only its ability to be easily worked on but also its mechanical performance and gasification rate which, on a broad spectrum, reposition it higher among other medical aids in short-term duration [22].

Tissue-engineering scaffolds (specifically for bone, cartilage, and skin) represent a significant avenue of research involving P3HB or PHBV, composites. These materials often incorporate ceramic fillers like β-tricalcium phosphate (β-TCP) or hydroxyapatite, which tend to enhance stiffness, osteoconductivity, and overall bioactivity. It is interesting to note that blending these composites with protein-based or polysaccharide elements can further promote cell adhesion. Studies conducted between 2020 and 2024, both in vitro and in vivo, generally show a boost in osteogenesis and bone mineral density when using PHB/PHBV-ceramic scaffolds and related blends [23,24,25]. When it comes to drug delivery and infection control, P3HB composites have been explored as vehicles for sustained release, for instance, to deliver antibiotics or anti-infective agents locally. Some hybrid nanocomposites, combining PHB or PHBV with fullerene or loaded with statins and antibiotics, have demonstrated promising control over release profiles. These approaches seem to support bone repair effectively while also targeting infection, which is a delicate balance in clinical scenarios [26,27]. In the areas of wound dressings, sutures, and soft-tissue devices, PHAs including P3HB have a long-standing history, particularly in the manufacture of sutures. Recent work has focused on developing non-woven fibrous mats, patches, and dressings, often by blending PHB with materials such as chitosan or polyurethane or creating electrospun mats. These composites improve cellular compatibility and help regulate degradation rates, which is an important factor for optimal wound healing [28,29]. Nerve conduits and guided tissue conduits also benefit from P3HB-based constructs. Reports on 3D-printed P3HB conduits, as well as fibre or tubular scaffolds, show their potential as guides for tissue regeneration and as carriers for mesenchymal stem cells (MSCs). Additive manufacturing techniques allow for detailed customization of geometry and porosity, which seems to be crucial for tailoring these conduits to specific tissue regeneration tasks [30,31]. Finally, the addition of nanofillers such as graphene oxide, cellulose nanocrystals (CNC), carbon nanotubes, or short natural fibres has been shown to strengthen P3HB composites significantly. This reinforcement not only enhances mechanical properties but also allows tuning of stiffness to better mimic different tissues—from the rigidity needed in bone implants to the flexibility required for soft tissues. Various studies in the early 2020s have documented improvements in mechanical behaviour and adjusted degradation rates, pointing toward their potential for long-term implantable devices [32,33].

One of the best improvements or changes that can be made to P3HB is the addition of nanocellulose, which can be attributed to properties such as thermal resistance, high strength and stiffness, as well as non-toxicity, biodegradability, and renewability [34,35]. Nanocellulose can be classified into three categories—cellulose nanocrystals (CNCs), cellulose nanofibers (CNFs), and bacterial nanocellulose (BC)—depending on its source and preparation method. These types of nanocellulose have a similar chemical composition but differ in physical properties—they vary in particle size, morphology, and crystallinity. CNCs are produced by chemical synthesis or extracted from plants by acid hydrolysis. They have a morphology resembling whiskers or a short rod, with a length of 100–500 nm and a diameter of 2–20 nm, and are characterised by high strength and high crystallinity. Mechanical methods extract CNFs. CNFs have crystalline and amorphous regions, and cellulose fibres have a length of 500–2000 nm and a diameter of 1–100 nm [36]. BC is the purest form of cellulose—unlike cellulose of plant origin, it does not contain hemicellulose, pectin and lignin. Non-potent aerobic bacteria produce bacterial cellulose. It is characterised by an ultra-thin network structure containing fibres of 80–150 nm in diameter, high crystallinity and elasticity, and high water retention capacity [37,38]. Nanocellulose, produced from renewable materials, can be easily incorporated with P3HB to provide mechanical benefits, increase flexibility, enhance thermal stability, and increase crystallinity [39]. The most recent studies have found that the addition of nanocellulose to P3HB composites leads to the increase in their tensile and elastic modulus, which, in the end, means the materials become more robust and fit for medical applications in need of high mechanical performance, such as tissue engineering scaffolds and sutures [22]. Additionally, a reinforced nanocellulose material can impart a smoother and more adhesive-like surface property to the material, making it a better substrate for biomedical applications such as tissue regeneration [40,41]. P3Hb composites with cellulose nanocrystals (CNCs) are characterised by high biocompatibility and non-toxicity, which makes them suitable for applications as drug carriers and wound dressing materials. Additionally, their biodegradation does not cause environmental acidification, which is beneficial in the context of their use as an implant [42,43].

The combination of P3HB and nanocellulose also enhances the material’s biodegradation rate, which is particularly important in medical applications, where the material should degrade over time as it fulfils its function in the body. The presence of nanocellulose in the polymer may lead to the polymer’s degradation, whereby P3HB decomposes as quickly as tissue repair [40]. Moreover, this composite material can be converted into a variety of forms, including films, scaffolds, and fibres, providing a broad range of options for creating medical devices with customised properties.

The study focused on creating biocomposites composed of P3HB filled with nanocellulose at concentrations of 0.5, 1, 2, and 3 weight%. It also aimed to analyse their thermal, mechanical, and biological properties. Thermal analysis was used to determine the phase contents of nanobiocomposites with varying filler amounts in the polymer matrix. Additionally, this study examined how the phase contents depend on the thermal history of the biocomposites. This represents our new method for analysing polymers and nanobiocomposites, as discussed in our earlier paper [44]. Thermal analysis results effectively predicted the mechanical properties. The nanobiocomposites produced were examined for homogeneity with scanning electron microscopy (SEM). Their thermal properties and phase compositions were analysed using differential scanning calorimetry (DSC). To establish the degradation temperature range of the composites, thermogravimetric analysis (TGA) was used. The mechanical properties of the obtained nanobiocomposites and the unfilled P3HB were tested, including tensile strength, elongation at break, impact strength by the Charpy method, and Shore hardness. The immunosafety of the tested nanocomposites and the absence of, or very low, endotoxin contamination was confirmed by the Food and Drug Agency Guidance, as well as the European Medicines Agency for further biomedical applications.

## 2. Results and Discussion

**Figure 1:**
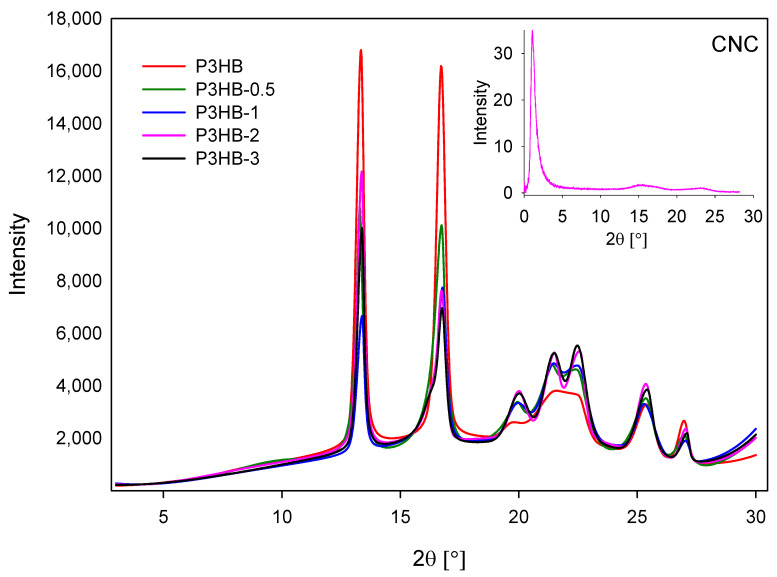
X-ray diffractograms recorded in transmission mode for unfilled P3HB, and composites containing 0.5 and 1, 2, and 3 wt.% nanocrystalline cellulose (designated P3HB-0.5, P3HB-1, P3HB-2, and P3HB-3, respectively). In the upper right corner, the diffraction pattern of the nanofiller (CNC) is shown.

Figure 1 shows X-ray diffractograms of the unfilled P3HB and its nanobiocomposites with nanocrystalline cellulose. The diffractograms indicate a gradient of increased resolution and peak sharpness with an increased amount of nanocrystalline cellulose in composites. It is observed that the peak resolution increased in the range 2Θ from 19 to 24°. It indicates the partial growth of crystallinity in the resulting nanocomposites, resulting from the increase in crystal number as the percentage of nanocrystalline cellulose rises. In the upper right corner of Figure 1, the diffraction pattern of the nanofiller is shown.

**Figure 2:**
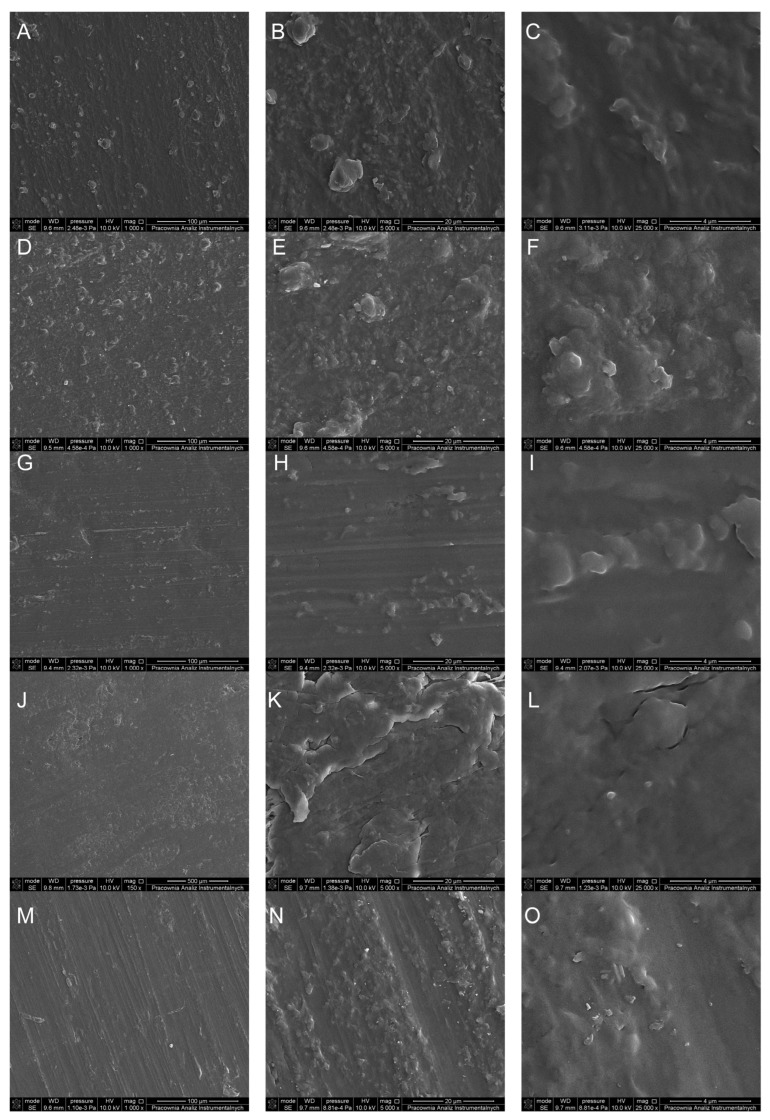
Scanning electron microscopy images of unfilled P3HB (**A**–**C**), and composites containing 0.5 and 1, 2, and 3 wt.% nanocrystalline cellulose (designated P3HB-0.5 (**D**–**F**), P3HB-1 (**G**–**I**), P3HB-2 (**J**–**L**), and P3HB-3 (**M**–**O**), respectively).

Scanning electron microscopy (SEM) images were captured to investigate the surface morphology of poly(3-hydroxybutyrate) (P3HB) and its nanocomposites containing varying concentrations of nanocrystalline cellulose. These micrographs were crucial in assessing the homogeneity and structural characteristics of the surfaces. The SEM image of the pristine P3HB surface depicted a homogeneous and pore-free morphology, as illustrated in Figure 2A–C. The P3HB composite with 0.5% nanocellulose (P3HB-0.5) mostly maintained a uniform surface, which indicates excellent dispersion of the nanocellulose in the polymer matrix (Figure 2D–F). The nanobiocomposites with 2% and 3% nanocellulose concentration displayed uniform and compact surface structures, as depicted in Figure 2J–O.

**Figure 3:**
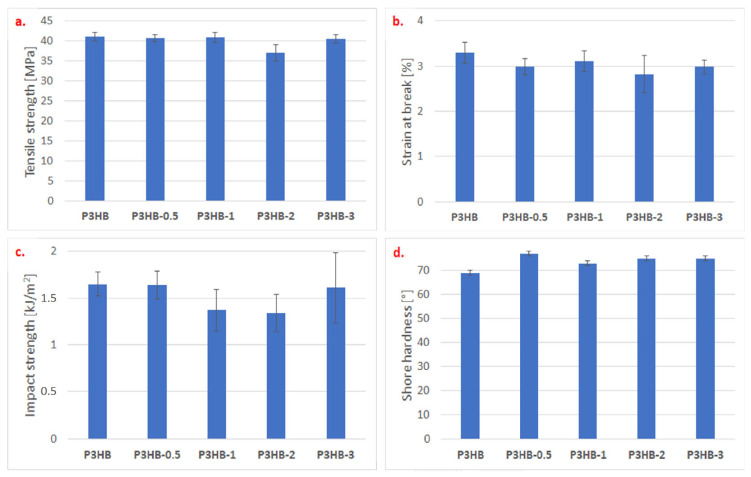
Effect of nanocrystalline cellulose content on the tensile strength (**a**), strain at break (**b**), impact strength (**c**), and Shore hardness (**d**) of P3HB and its nanocomposites containing 0.5 and 1, 2, and 3 wt.% (designated P3HB-0.5, P3HB-1, P3HB-2, and P3HB-3, respectively).

The influence of the addition of nanocrystalline cellulose on the mechanical properties of the produced nanocomposites based on the P3HB matrix was also investigated. Figure 3 present test results as mean ± SD. Statistical analysis used the nonparametric Mann–Whitney U test, with significance at *p* < 0.05. The tensile strength of the obtained nanocomposites was tested, and the presence of nanofiller had a slight influence on the change in tensile strength (Figure 3a). The results of the statistical analysis indicate significant differences exist between P3HB-2 and all other nanocomposites. No significant differences were found among P3HB, P3HB-0.5, P3HB-1, and P3HB-3. The largest difference is between P3HB-2 and P3HB. The presence of crystalline nanocellulose caused a minimal decrease in tensile strength, except for the addition of 2% by weight of nanocellulose, where a reduction of 10% was observed compared to the unfilled P3HB. Similarly, a decrease in the strain at break was observed (Figure 3b). P3HB differs significantly from P3HB-0.5, P3HB-2, and P3HB-3. The largest difference is between P3HB and P3HB-2 (*p* = 0.008). Non-homogeneous crystalline areas or a rigid amorphous fraction may probably cause the dependence. Agglomerates may cause a decrease in parameters. After adding the nanoadditive, the strain at break decreased. The value fluctuated by 10% and remained around 3%, regardless of the amount of nanocellulose.

In the case of testing the impact strength of the produced nanocomposites (Figure 3c), it was noticeable that the impact strength decreased with the increase in the nanocellulose content from 0.5 to 2% by weight. P3HB differs significantly from P3HB-1 and P3HB-2. The largest difference is between P3HB-0.5 and P3HB-2 (*p* = 0.016). This suggests a stiffening effect of the nanoadditive. However, the use of 3 wt.% nanocellulose caused a renewed increase in impact strength, almost reaching the impact value of the unfilled P3HB. Moreover, an apparent increase in the hardness of the produced nanocomposites (Figure 3d) by an average of 10% was observed, indicating a stiffening of the material’s structure. P3HB differs significantly from all other nanocomposites. The largest difference is between P3HB and P3HB-0.5 (*p* = 0.008).

The likely cause of the improved mechanical properties is the interaction of the nanofiller with the polymer in the amorphous zone, for example, through the formation of hydrogen bonds. This results in lower strain at break and impact strength, as well as increased hardness. This also confirms the nanofiller’s lack of effect on tensile strength, which may be attributed to its insufficient content in the polymer to achieve the appropriate level of interaction. This research will be continued in our future work.

**Figure 4:**
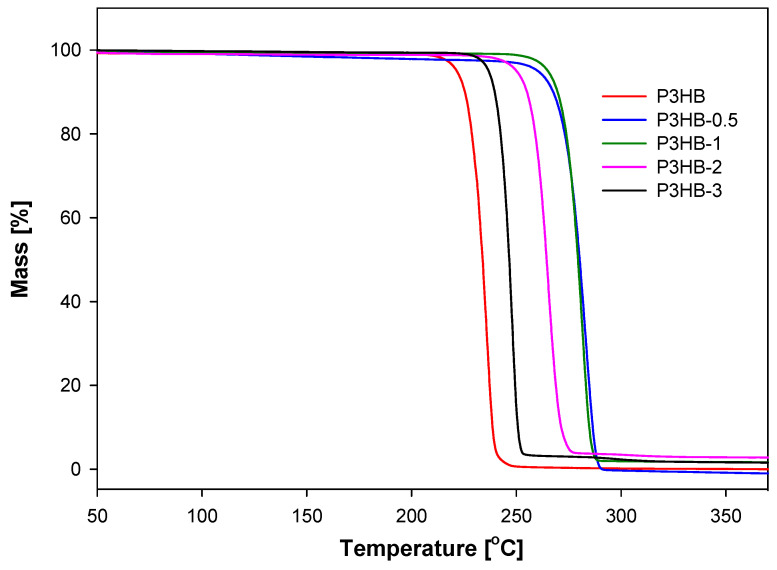
TG analysis of the unfilled P3HB and their nanocomposites containing 0.5, 1, 2, and 3 mass/% of nanocrystalline cellulose (they were marked as P3HB-0.5, P3HB-1, P3HB-2, and P3HB-3, respectively).

To examine the durability and processing and functional properties of the obtained nanomaterials, a thermogravimetric analysis (TGA) was carried out (Figure 4). The TG curves of the nanocomposites indicate that their thermal stability exceeds that of unfilled P3HB. Adding nanocrystalline cellulose to the P3HB matrix raises the degradation temperature of the nanocomposites. For example, a 2% mass loss occurs at 222.8 °C. For the obtained nanocomposites, the degradation temperatures reported under the same conditions were 248.6, 248.1, 232.1, and 220.0 °C for P3HB-0.5, P3HB-1, P3HB-2, and P3HB-3, respectively. The weight loss temperature drops as the nanocrystalline cellulose content in the polymer matrix increases. Adding 0.5% or 1% nanofiller raises the degradation temperature of the nanocomposites by approximately 26 °C compared to the unfilled P3HB.

**Figure 5:**
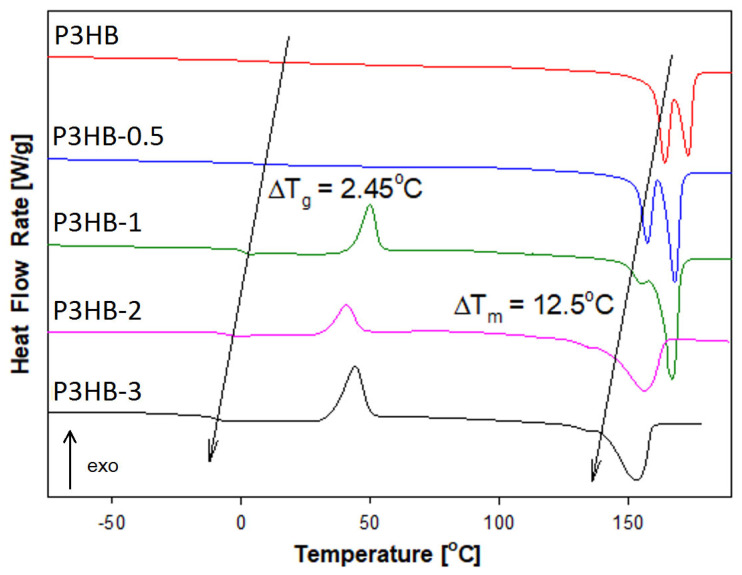
Heat flow rate vs. temperature obtained based on the DSC analysis of the unfilled P3HB and its nanocomposites containing 0.5, 1, 2 and 3 mass % of nanocrystalline cellulose (they were marked as P3HB-0.5, P3HB-1, P3HB-2, P3HB-3, respectively). The results were obtained during heating at a rate of 10 °C/min after cooling the sample at a constant rate of 10 °C/min from the melt.

The thermal properties of the obtained composites were also examined using standard differential scanning calorimetry (DSC). Figure 5 presents a comparison of the heat flow rate versus temperature for P3HB and nanocomposites with 0.5, 1, 2, and 3 wt.% filler. These measurements were taken with a heating rate of 10 °C/min over a temperature range from −90 °C to 195 °C, after cooling the samples at the same rate from 195 °C back to −90 °C.

The qualitative thermal analysis was performed by examining the heat flow rate of semicrystalline P3HB and its nanobiocomposites. During heating, all materials showed a glass transition and melting. For P3HB-1, P3HB-2, and P3HB-3, cold crystallisation occurred between these two regions (see Figure 5). Thermal phase transition parameters were estimated and listed in Table 1. The change in heat capacity at the glass transition, ΔC_p_, and T_g_ were determined from the glass transition region during heating. The heat of fusion, ΔH_f_, and melting temperatures T_m1_ and T_m2_ were derived from the melting region analysis. The molar masses of P3HB and its nanobiocomposites P3HB-0.5, P3HB-1, P3HB-2, and P3HB-3 were calculated as 86.09 g/mol, 86.32 g/mol, 86.47 g/mol, 86.92 g/mol, and 87.3 g/mol, respectively. Notably, the ΔC_p_ values are smaller, and the ΔH_f_ values are larger, for the nanobiocomposites compared to pure P3HB. Additionally, the dual melting peaks observed in the analysis of P3HB persisted in the nanobiocomposites, likely due to the presence of crystals with different lamella thicknesses [44,45].

The study examined how adding nanocrystalline cellulose affects the glass transition temperature (T_g_), melting temperature (T_m_), heat of fusion, and heat capacity at T_g_. Figure 5 displays the curves for unfilled P3HB and its nanobiocomposites with different nanofiller levels. It is evident that both T_g_ and T_m_ decrease as the nanofiller content increases.

The variation in glass transition temperature for the nanocomposite is based on the highest amount of nanofiller in comparison with the unfilled P3HB, which equals 2.5 °C. The change in melting temperature between P3HB and P3HB-3 reached 12.5 °C. These relationships confirm the good dispersion of nanofiller in the polymeric matrix as reported earlier [46]. The decrease in the glass transition temperature of the nanobiocomposite, compared to unfilled P3HB, with increasing amounts of introduced nanofiller indicates the plasticizing effect of the nanocrystalline cellulose in reference to unfilled P3HB.

**Figure 6:**
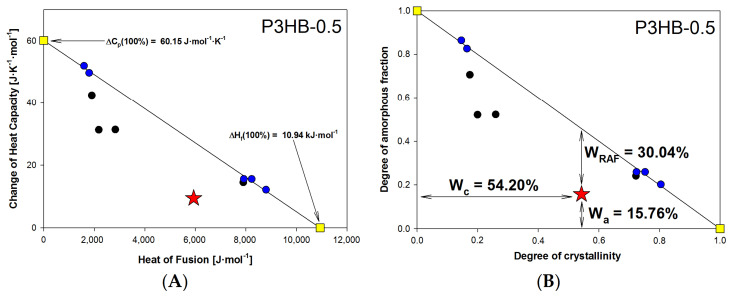
(**A**) The heat capacity change at the glass transition temperature on the heat of fusion of the P3HB-0.5 nanobiocomposite; (**B**) The dependence of the amorphous fraction as a function of the degree of crystallinity of the semicrystalline P3HB-0.5 nanobiocomposite.

The thermal history of nanobiocomposites was also examined. Changes in heat capacity at T_g_ related to the heat of fusion, obtained at different cooling rates (1–20 °C/min), are shown graphically in Figure 6A,B. The P3HB matrix was previously studied in our earlier work [44]. It is important to highlight that changes in heat capacity and other phase transition parameters were determined using qualitative thermal analysis, unlike the more common quantitative thermal analysis documented extensively in the literature [44,46,47].

Most semicrystalline polymer materials in the solid state consist of three phases: the crystalline phase (c), the mobile amorphous fraction (a), and the rigid amorphous fraction (RAF) [44]. For semicrystalline materials that only have two phases—the mobile amorphous phase and the crystalline phase—the plot showing the change in heat capacity at T_g_ against the heat of fusion displays a linear trend. The experimental measurements are represented by the blue and black circles, as well as the red star. The solid straight line results from fitting this data, which spans from the fully amorphous state, ΔC_p_ (100%), to the fully crystalline state, ΔH_f_ (100%), for P3HB-0.5 (with ΔH_f_ (100%) marked by yellow squares).

The change in heat capacity at T_g_ for fully amorphous P3HB-0.5 was estimated at 60.15 J·mol^−1^·K^−1^, while the heat of fusion for fully crystalline P3HB-0.5 was 10.94 kJ/mol. The experimental points forming the solid straight line are characteristic of a two-phase model. Points outside this line (red star and black circles) suggest a three-phase model. In Figure 6B, the relationship between the amorphous phase degree, W_a_, and the crystallinity degree, W_c_, of the P3HB-0.5 nanobiocomposite is shown. Data from Figure 6A were recalculated to W_a_ and W_c_ using W_a_ = ΔC_p_/ΔC_p_ (100%), and W_c_ = ΔH_f_/ΔH_f_ (100%). The rigid amorphous fraction, W_RAF_, was estimated as W_RAF_ = 1 − W_a_ − W_c_, based on reference [44]. All equilibrium parameters for the P3HB nanobiocomposites are listed in Table 2, along with reference data collected from [44].

Table 2 presents the calculated phase contents for samples cooled at 10 °C/min and then heated at the same rate. The results show that adding nanocrystalline cellulose between 0.5% and 2% reduces the nanobiocomposites’ degree of crystallinity. A small nanofiller content (0.5–2%) enhances the properties of the nanocomposite. However, higher filler amounts, such as in P3HB-3, may lead to nanoparticle agglomeration, adversely affecting the material’s properties.

**Figure 7:**
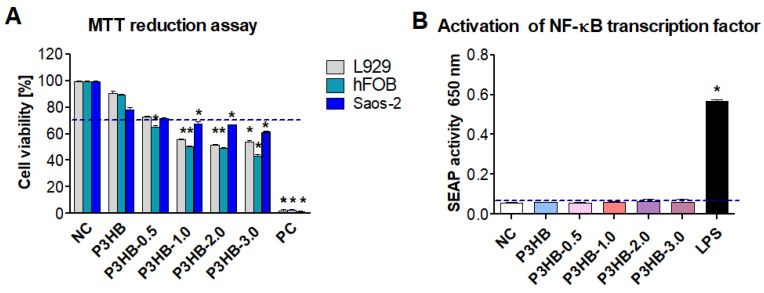
The biocompatibility and immunocompatibility of P3HB, P3HB-0.5, P3HB-1, P3HB-2, and P3HB-3 were evaluated. (**A**) The cytotoxicity of these materials towards mouse L929 cells, as well as human hFob 1.19 and Saos-2 cells, was assessed by measuring the percentage of cells capable of reducing the tetrazolium salt (3-(4,5-dimethylthiazol-2-yl)-2,5-diphenyltetrazolium bromide, or MTT). NC refers to negative control cells treated with H_2_O_2_, while PC refers to the positive control, which consists of cells in medium alone. Results are presented as mean ± standard deviation (SD). Each experimental variant was tested in three separate experiments, with triplicate samples for each. The red line marks the threshold of 70% viable cells, the minimum percentage needed to consider the extracts non-cytotoxic in vitro. Statistical significance was assessed using the nonparametric Mann–Whitney U test, with *p* < 0.05 indicating significance (*), by comparing unstimulated and stimulated cells. (**B**) Activation of embryonic alkaline phosphatase (SEAP) in THP-1 -Blue monocytes was evaluated. These cells were stimulated with either lipopolysaccharide (LPS) from *E. coli* or various P3HB concentrations (P3HB, P3HB-0.5, P3HB-1, P3HB-2, P3HB-3). Control cells were cultured in medium alone. Supernatants were collected and assessed for SEAP activity via colourimetry. The experiments were conducted three times in triplicate for each condition. Results are presented as mean ± SD. Statistical analysis used the nonparametric Mann–Whitney U test, with significance at *p* < 0.05 (*: unstimulated versus stimulated cells).

To explore potential applications of the newly developed nanocomposites, we also examined cell viability and immunocompatibility with the tested materials. The effects of P3HB, P3HB-0.5, P3HB-1, P3HB-2, and P3HB-3 on the viability of mouse fibroblasts (reference L929) and human cell lines hFBO 1.19 and Saos-2 were evaluated using the MTT reduction assay. This method assesses mitochondrial dehydrogenase activity in the presence and absence of the tested substances. In this assay, higher mitochondrial enzyme activity indicates greater cell viability. The viability of L929, hFOB 1.19, and Saos-2 cells incubated with P3HB, P3HB-0.5, and other tested lines was greater than 70%, confirming that they meet the biological safety standards (see Figure 7A). However, for biomaterials like L929 and the hfob 1.19 cell line, P3HB-0.5, P3HB-1.0, P3HB-2.0, and P3HB-3.0 did not meet biosafety criteria as cell viability was below 70% (Figure 7A). Monocytes are highly responsive cells that quickly initiate an inflammatory response upon activation. Effective healing depends on controlled inflammation, which promotes revascularisation and tissue regeneration. However, excessive activation of monocytes—caused by biomaterial components or contaminants, such as endotoxins—can lead to acute inflammation, potentially resulting in pus formation, tissue damage, and breakdown of cell barriers [48]. THP1-Blue™ cells, similar to other monocytes, respond to ligands such as peptides, endotoxins, and glycoconjugates via toll-like receptors (TLRS): TLR-2, TLR-4, TLR-5, TLR-6, and TLR-8. When these TLRS are stimulated, NF-κB is activated, leading to the secretion of the SEAP enzymatic marker into the cell culture medium. Our study demonstrates that THP-1xBlue monocytes respond to LPS by producing high levels of SEAP, indicating the activation of the nuclear transcription factor NF-κB.

The compounds P3HB, P3HB-0.5, P3HB-1, P3HB-2, and P3HB-3 did not induce SEAP activity at any tested concentration, even at concentrations higher than those of the negative control, which involved cells grown in medium alone. This confirms the immunosafety of the extracts and suggests a lack of or very low endotoxin contamination. According to guidelines from the Food and Drug Administration and the European Medicines Agency, endotoxin levels in biomaterials that come into contact with human blood or tissue should not exceed 0.25 EU. Our optimisation study showed that activating THP1-Blue™ monocytes with 0.25 EU of endotoxin (the positive control) caused a significant increase in SEAP production, with absorbance reaching 1.5 ± 0.02 at 620 nm. (Figure 7B).

**Figure 8:**
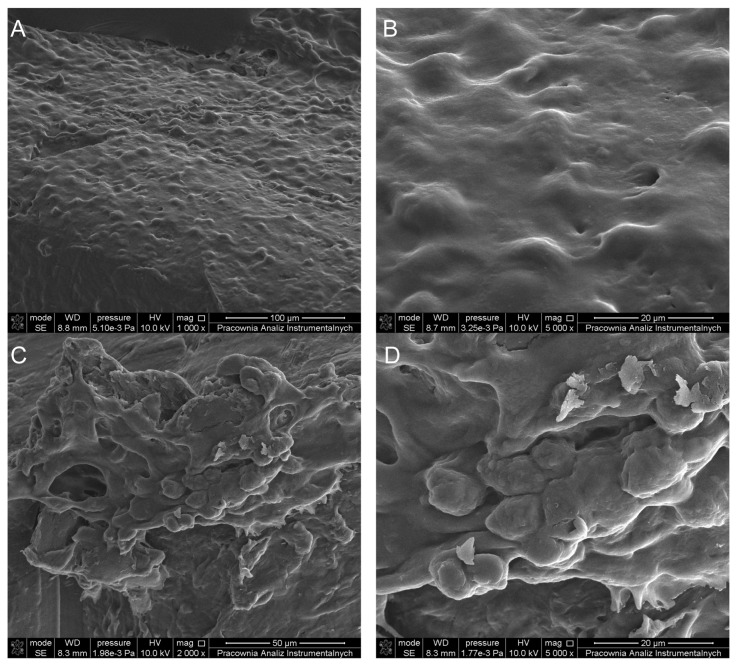
SEM micrographs of L929 cells cultured on P3HB (**A**,**B**) and its nanocomposite containing 0.5 wt.% (P3HB-0.5) (**C**,**D**) after 2 days of culture.

Comparative images from Scanning Electron Microscopy (SEM) revealed the morphological characteristics and proliferation rates of L929 fibroblasts, as shown in Figure 8. The obtained results indicate that L929 cells adhere to the surface of P3HB and can proliferate. Morphology of L929 cells exhibited typical structural characteristics. This suggests that under the given conditions, the cells maintained a morphology consistent with healthy, unaltered L929 fibroblast cells. Cells had a regular shape, size, and appearance characteristic of fibroblasts, without signs of damage or abnormality (Figure 8A,B). Similarly, for P3HB-0.5 (Figure 8C,D), the surface enabled the adhesion and division of L929 fibroblasts, which confirms the results obtained from the cytotoxicity tests.

## 3. Materials and Methods

### 3.1. Materials

Poly(3-hydroxybutyrate), P3HB, was obtained from Biomer (Krailling, Germany). Its weight average molecular weight (M_w_) is 443,900 g/mol, with a dispersity (M_w_·M_n_^−1^) of 5.72, determined by size exclusion chromatography in chloroform. The melt flow index of P3HB is 0.11 g·(10 min)^−1^ at 180 °C under 2.16 kg load. The molar mass of its repeating unit is 86.09 g/mol.

Nanocrystalline cellulose (NCC), supplied by Nanografi Nanotechnology (Ankara, Turkey), has a CAS number of 9004-34-6. Its particle size ranges from 10 to 20 nm in diameter to 300–900 nm in length. The average density is 1.49 g/cm^3^. The decomposition temperature (TGA in N2) is 349 °C, and its crystallinity (assessed by XRD) is 92%.

### 3.2. Methods and Instrumentation

#### 3.2.1. Nanobiocomposites Preparation

Nanocomposites were produced by melt mixing P3HB with varying nanocrystalline cellulose contents—0.5, 1, 2, and 3 mass %—using a co-rotating twin-screw micro-extruder. Before extrusion, the nanofiller was homogenised with the polymer matrix in an electric grinder. The melt mixing conditions and complete characterisation of the P3HB and nanocrystalline cellulose nanocomposites are detailed in Table 3. Additionally, unfilled P3HB was melt mixed to serve as a control sample. The sample abbreviations and nanocrystalline cellulose content are also provided in Table 3.

#### 3.2.2. Thermogravimetry

Thermogravimetric analysis (TGA) was performed using a Mettler Toledo (Greifensee, Switzerland) TGA/DSC 3+ instrument. Samples were heated from 25 to 600 °C at a rate of 5 °C/min in a nitrogen atmosphere.

#### 3.2.3. Differential Scanning Calorimetry

All calorimetric experiments were conducted with a differential scanning calorimeter (DSC), Q1000TM, from TA Instruments, Inc. (New Castle, DE, USA). This heat-flux type calorimeter features a mechanical refrigerator to regulate the heating and cooling of samples. Measurements were performed in a nitrogen atmosphere at a steady flow rate of approximately 50 mL/min. The DSC samples weighed around 10 mg. Standard DSC measurements covered a temperature range of −90 to 195 °C, with a variable cooling rate of 1–40 °C/min and a fixed heating rate of 10 °C/min. An isothermal annealing step was performed for 2 min at 195 °C, followed by stabilisation for 5 min at −90 °C. Calibration of temperature and heat flow was performed using melting parameters of indium, with an onset melting temperature of T_m_(onset) = 156.6 °C and a heat of fusion of ΔH_f_ = 28.45 J/g (3.281 kJ/mol). To ensure accuracy, the heat capacity was calibrated with sapphire. The heat flow data was collected during the second heating cycle after controlled cooling.

#### 3.2.4. X-Rays

The phase composition of the samples was determined using a Rigaku (The Woodlands, TX, USA) Miniflex II X-ray diffractometer. A filtered copper lamp (CuKα, λ = 0.1542 nm) operating at a 40 kV voltage was used. The measurement range was 2θ = 3–30°, with a step size of 0.02° per 3 s.

#### 3.2.5. Scanning Electron Microscopy

The surface morphology of unfilled P3HB and nanobiocomposites with 0.5, 1, 2, and 3 wt.% nanocrystalline cellulose was examined using high-resolution scanning electron microscopy. Prior to SEM analysis, the samples were sputter-coated with a nanometric conductive layer of gold/palladium (8:2) using a SC7620 sputter coater (Quorum Technologies, Lewes, UK) to minimise charging effects and enhance image quality. SEM imaging was carried out on a Quanta 3D FEG instrument (FEI, Hillsboro, OR, USA) at an accelerating voltage of 10 kV.

#### 3.2.6. Mechanical Tests

The mechanical properties of the obtained nanobiocomposites and unfilled P3HB were evaluated, including tensile strength ($\sigma_{M}=\frac{F_{M}}{A_{0}}$; A_0_—cross-section of the sample [mm^2^]; F_M_—maximum value of tensile force [N]), elongation at break ($\varepsilon_{M}=100\times \frac{\Delta L_{0}}{L_{0}}$, L_0_—length of the measuring section [mm]; ΔL_0_—change in the length of the test piece between the measurement marks [mm]); impact strength (measured using the Charpy method; KCU = E/A_0_; E—the energy needed to break the sample [J]; A—cross-section [mm^2^]), and Shore hardness. Tensile strength and strain at break were measured following the standard [PN-EN ISO 527-1:2020-01] [49], with tests conducted at a jaw feed rate of 50 mm/min. Charpy impact tests on notched samples were performed using the IMPats-15/50, ATSFAAR S. p. A. hammer, according to [PN-EN ISO 179-1:2010] [50]. Shore hardness measurements were carried out according to [PN-ISO 868:1998], utilising Zwick Roell equipment [51], with the scale D used.

#### 3.2.7. Cell Culture and In Vitro Biocompatibility Assessment

Reference L929 mouse fibroblasts and human Hs68 skin fibroblasts were used to evaluate biocompatibility in vitro. Mouse fibroblasts were cultured at 37 °C with 5% CO_2_ in RPMI-1640 medium (Biowest, Nuaillé, France), with 10% FBS and antibiotics: penicillin and streptomycin. Human foetal osteoblastic hFOB 1.19 cells, obtained from ATCC, were grown in a 1:1 mix of phenol-free Dulbecco’s and Ham’s 12-F media (Gibco, Waltham, MA, USA), with 2.5 mL l-glutamine, 10% FBS, and 0.3 mg/mL geneticin. Human osteosarcoma Saos-2 cells, from ATCC, were cultured in McCoy’s 5A Medium with 15% FBS and antibiotics. Cultures were maintained in logarithmic growth with medium changes performed two or three times weekly, and passaged using 0.25% trypsin-EDTA.

Cytotoxicity of P3HB, P3HB-0.5, P3HB-1, P3HB-2, and P3HB-3 was evaluated on hFOB 1.19 and Saos-2 cells (2 × 10^5^ cells/mL) following ISO standard 10993-5 (2009) and the MTT assay [52]. Each sample covered one-tenth of the well, with cells in medium serving as a positive control and 0.03% H_2_O_2_ as a negative control. Absorbance at 570 nm was measured in triplicate, and cytotoxicity was expressed as the percentage of MTT reduction relative to controls. Statistical significance was determined at *p* < 0.05 using the Mann–Whitney test, with data presented as mean ± SD, analysed with STATISTICA 12.3 PL.

For microscopic analysis, a density of 2.5 × 10^4^ cells/mL of L929 cells was cultured on the tested polymers. The cultures were maintained at 37 °C with 5% (*v*/*v*) CO_2_ for 2 days. Before seeding, the polymers were sterilised with 70% ethanol and exposed to UV light. Cells that adhered were fixed with 2.5% glutaraldehyde in PBS for 30 min, then rinsed three times for 5 min each in PBS and increasing concentrations of ethanol. The dehydration process involved a 2 min step in 95% ethanol, followed by four 5 min steps in 100% ethanol. Cells were further dehydrated twice for 10 min each using 100% hexamethyldisilazane (HMDS) at room temperature. The polymer samples with adhered cells were mounted on a holder with carbon tape, then coated with gold using a SC7620 Mini Sputter Coater (Quorum Technologies, Lewes, UK), and examined with a Quanta 3D FEG scanning electron microscope/focused ion beam (SEM/FIB).

#### 3.2.8. Monocyte Activation Assay

THP1-Blue™ cells (Invitrogen, Waltham, MA, USA), derived from the human THP-1 monocyte line, were used to test whether P3HB, P3HB-0.5, P3HB-1, P3HB-2, and P3HB-3 could activate monocytes by inducing nuclear factor kappa B (NF-κB). These cells, engineered from THP-1, stably contain an NF-κb-responsive SEAP reporter. NF-κB activation causes SEAP secretion, which is detectable spectrophotometrically using Quanti-Blue. Cells were cultured at 37 °C with 5% CO_2_ in RPMI-1640 with 10% FBS, HEPES, penicillin/streptomycin, glutamine, and blastidin. For activation assays, 5 × 10^5^ cells/mL of THP-1 Blue™ were stimulated with P3HB variants or controls (medium alone as negatives; LPS as positive). SEAP activity was measured at 650 nm using a plate reader. All experiments were performed in triplicate; significance was assessed at *p* < 0.05 using the U Mann–Whitney test, and data are presented as mean ± SD. Graphs were generated with GraphPad Prism 10.0 software (https://www.graphpad.com/, GraphPad Software Inc., San Diego, CA, USA). Data are presented as mean ± standard deviation (SD). Group differences were evaluated using the non-parametric Mann–Whitney U test or the Kruskal–Wallis one-way ANOVA. Statistical analysis was performed with Statistica 13 PL software (https://statistica.software.informer.com/13.3, accessed on 28 September 2025). Results were deemed statistically significant at *p*  <  0.05. The Shapiro–Wilk test (S–W) was employed to assess the normality of data distribution.

## 4. Conclusions

Nanocomposites of P3HB and nanocrystalline cellulose were produced by directly mixing them in a co-rotating twin-screw micro-extruder. The results clearly demonstrated how the nanofiller in the P3HB matrix affects its thermal, mechanical, and biological properties.

D’Arienzo et al. (2024) [53] investigated the feasibility of using cellulose fibre–reinforced polyhydroxybutyrate (PHB) composites in additive manufacturing. The aim was to create sustainable, biodegradable filaments suitable for 3D printing. The results showed that low levels of cellulose reinforcement (1.5–3 wt.%) are compatible with existing fused deposition modelling processes, producing stiffer and more environmentally friendly PHB filaments. Researchers conclude, that optimising cellulose dispersion and treatment could enhance reinforcement efficiency and maintain toughness.

Panaitescu et al. [54] developed and characterised biodegradable thin films composed of three biopolymers-PLA (poly (lactic acid)), PHB (polyhydroxybutyrate), and PHBV (polyhydroxybutyrate-co-valerate)-each reinforced with cellulose nanocrystals (CNCs). Observed, that CNC incorporation and C-SVA altered film morphology significantly. Large crystalline formations observed included. CNC acted as effective nucleation agents during solvent vapour annealing, triggering the development of distinct crystalline patterns and altering the film microstructure, providing a sustainable pathway for creating biodegradable, microstructured polymer films.

The results are consistent with ours. However, no researcher has yet determined the following parameters: the enthalpy of crystalline nanocomposites based on P3HB and nanocellulose, and the change in heat capacity of a completely amorphous material, which would allow for an accurate analysis of the phase content and bring closer the possibilities of conducting advanced thermal analysis in the future.

Introducing nanocrystalline cellulose into a polymer matrix enhances the thermal stability of the nanocomposites, as shown by TG measurements under non-oxidative conditions. The best thermal stability was observed for P3HB-0.5 and P3HB-1, which was comparable to the degradation temperature value of 2% weight loss. The thermal analysis revealed that increasing the nanofiller content in the P3HB composites led to a decrease in both the glass transition and melting temperatures. A lower melting point is advantageous because it broadens the processing window, enabling easier manufacturing of the nanocomposites compared to unfilled P3HB. The optimal thermal properties and the widest processing window—measured as the temperature difference between degradation and melting—were observed in P3HB-1 and P3HB-0.5, with values of 81.5 °C and 80.7 °C, respectively. This movement, apart from the melting and decomposition temperatures of nanocomposites based on P3HB, facilitates processing and prevents the degradation of the material.

Adding nanocrystalline cellulose to the P3HB matrix reduces the crystallinity degree in P3HB-0.5, P3HB-1, and P3HB-2 nanobiocomposites. X-ray spectra indicate an increase in crystallinity for P3HB-3, likely due to a higher nanocrystalline cellulose content leading to an increased number of crystals.

On the basis of the mechanical properties, it can be concluded that the addition of nanocellulose reduces the polymer matrix’s ability to deform, particularly in the amorphous zone of the polymer, resulting in increased stiffness of the resulting nanocomposites. DSC studies have shown that the addition of nanocellulose reduces the degree of polymer crystallinity. Mechanical and thermal tests showed that the most desirable properties oscillate around P3HB-0.5 and P3HB-1, indicating the advantage of P3HB-1, which displays the most plastic character in relation to P3HB. The presence of crystalline nanocellulose caused a minimal decrease in tensile strength, except for the addition of 2% by weight of nanocellulose, where a reduction of 10% was observed compared to the unfilled P3HB. Similarly, a reduction in the strain at break was observed. Non-homogeneous crystalline areas or a rigid amorphous fraction may probably cause the dependence. Agglomerates may cause a decrease in parameters. However, biological studies regarding implant applications have shown that only P3HB-0.5 can be of key importance. The Food and Drug Agency Guidance and the European Medicines Agency confirmed the immunosafety of the tested extracts and the absence or very low levels of endotoxin contamination.

## Figures and Tables

**Table 1:** Comparison of the thermal parameters of the P3HB composites with nanocomposites of representative samples obtained while heating at a rate of 10 °C∙min^−1^ after cooling at the same rate.

Name of Sample	T_g_ [°C]	ΔC_p_ [J·mol^−1^·°C^−1^]	T_m1_ [°C]	T_m2_ [°C]	ΔH_f_ [kJ·mol^−1^]
P3HB	9.8	8.3	-	165.9	9.5
P3HB-0.5	7.4	9.48	157.3	167.9	5.93
P3HB-1	0.6	34.51	155.5	166.6	2.71
P3HB-2	−5.7	34.98	134.4	156.3	2.04
P3HB-3	−10.2	12.22	134.2	153.3	7.44

**Table 2:** Comparison of the phase content of P3HB, P3HB-0.5, P3HB-1, P3HB-2, and P3HB-3 representative samples obtained by heating at a rate of 10 °C∙min^−1^ after cooling at the same rate.

Name of Sample	W_a_ [%]	ΔC_p_ (100%) [J·mol^−1^·°C^−1^]	W_c_ [%]	ΔH_f_ (100%) [kJ·mol^−1^]	W_RAF_ [%]
P3HB	20.24	41.00	76.00	12.5	3.76
P3HB-0.5	15.76	60.15	54.20	10.94	30.04
P3HB-1	64.05	53.88	23.14	11.71	12.81
P3HB-2	62.59	55.89	17.88	11.41	19.53
P3HB-3	24.95	48.97	71.40	10.42	3.65

**Table 3:** Sample abbreviations and preparation conditions of obtained nanocomposites.

Sample Designation	Content of Nanocrystalline Cellulose	Temperature of Extrusion	Speed of Rotation
P3HB	0	Zone 1: 125 °C	350 rpm
P3HB-0.5	0.5	Zone 2: 135 °C	
P3HB-1	1	Zone 3: 150 °C	
P3HB-2	2	Zone 4: 160 °C	
P3HB-3	3	Head: 166 °C	

## Data Availability

The data will be made available on request.
